# Sustained Weight Loss with Vagal Nerve Blockade but Not with Sham: 18-Month Results of the ReCharge Trial

**DOI:** 10.1155/2015/365604

**Published:** 2015-07-12

**Authors:** Scott A. Shikora, Bruce M. Wolfe, Caroline M. Apovian, Mehran Anvari, David B. Sarwer, Robert D. Gibbons, Sayeed Ikramuddin, Christopher J. Miller, Mark B. Knudson, Katherine S. Tweden, Michael G. Sarr, Charles J. Billington

**Affiliations:** ^1^Division of General and Gastrointestinal Surgery, Brigham and Women's Hospital, 75 Francis Street, Boston, MA 02115, USA; ^2^EnteroMedics Inc., 2800 Patton Road, St. Paul, MN 55113, USA; ^3^Department of Surgery, Oregon Health & Science University, 3181 SW Sam Jackson Park Road, Portland, OR 97239, USA; ^4^Department of Medicine, Section of Endocrinology, Diabetes and Nutrition, Boston University School of Medicine, 88 East Newton Street, Boston, MA 02118, USA; ^5^Department of Surgery, McMaster University, 1280 Main Street West, Hamilton, ON, Canada L8S4L8; ^6^Departments of Psychiatry and Surgery, Perelman School of Medicine, Hospital of the University of Pennsylvania, 3535 Market Street, Philadelphia, PA 19104, USA; ^7^Departments of Medicine and Public Health Sciences, University of Chicago, 5841 S. Maryland Avenue, Chicago, IL 60637, USA; ^8^Department of Surgery, University of Minnesota, University of Minnesota Medical Center, 420 Delaware Street SE, Minneapolis, MN 55455, USA; ^9^3D Communications LLC, 8386 Six Forks Road, Raleigh, NC 27615, USA; ^10^Department of Gastroenterologic and General Surgery, Mayo Clinic Rochester, 200 First Street SW, Rochester, MN 55905, USA; ^11^Division of Endocrinology and Diabetes, University of Minnesota, Minneapolis, Minnesota Veterans' Administration Medical Center, One Veterans Drive, Minneapolis, MN 55417, USA

## Abstract

*Background/Objectives*. Vagal block therapy (vBloc) is effective for moderate to severe obesity at one year. *Subjects/Methods*. The ReCharge trial is a double-blind, randomized controlled clinical trial of 239 participants with body mass index (BMI) of 40 to 45 kg/m or 35 to 40 kg/m with one or more obesity-related conditions. Interventions were implantation of either vBloc or Sham devices and weight management counseling. Mixed models assessed percent excess weight loss (%EWL) and total weight loss (%TWL) in intent-to-treat analyses. At 18 months, 142 (88%) vBloc and 64 (83%) Sham patients remained enrolled in the study. *Results*. 18-month weight loss was 23% EWL (8.8% TWL) for vBloc and 10% EWL (3.8% TWL) for Sham (*P* < 0.0001). vBloc patients largely maintained 12-month weight loss of 26% EWL (9.7% TWL). Sham regained over 40% of the 17% EWL (6.4% TWL) by 18 months. Most weight regain preceded unblinding. Common adverse events of vBloc through 18 months were heartburn/dyspepsia and abdominal pain; 98% of events were reported as mild or moderate and 79% had resolved. *Conclusions*. Weight loss with vBloc was sustained through 18 months, while Sham regained weight between 12 and 18 months. vBloc is effective with a low rate of serious complications.

## 1. Introduction

Vagal nerve blockade (vBloc) has been recently studied as a minimally invasive laparoscopic surgery for weight loss and improvement in weight-related conditions such as type 2 diabetes mellitus (DM2) [1–3]. The most frequently performed bariatric surgical procedures for weight loss, Roux-en-Y gastric bypass and sleeve gastrectomy, produce considerable weight loss but have potentially serious complications and require alterations of the gastrointestinal (GI) anatomy that are not acceptable to many patients [4]. The vBloc device was developed for patients with moderate to severe obesity, as an alternative to conventional weight loss surgery and does not require permanent anatomical alteration.

Two recent studies have examined the effect of vBloc on weight loss with a rechargeable device. The single-arm, prospective vBloc DM2 study showed that at one year subjects with DM2 and body mass index (BMI) between 30 and 40 kg/m^2^ achieved 25% excess weight loss (%EWL) [2]. In the double-blind, randomized ReCharge trial, using the last-observation-carried-forward (LOCF) analysis method, the vBloc arm achieved 24% EWL (9.2% total weight loss [%TWL]) at 12 months, which was significantly greater than the 16% EWL (6.0% TWL) achieved by subjects in the Sham arm implanted with a Sham neuroregulator [1]. Both studies showed that vBloc had a low rate of serious device-related complications in the first year. This report summarizes additional safety and efficacy data from the ReCharge trial beyond 12 months as patients were unblinded through 18 months of follow-up.

## 2. Methods

### 2.1. Participants

The methods of the ReCharge trial have been described previously [1]. Briefly, participants were enrolled at 8 sites in the United States and 2 sites in Australia. Participants were eligible for inclusion if their BMI was between 35 and 40 kg/m^2^ with one or more obesity-related comorbidities (i.e., type 2 diabetes mellitus, hypertension, dyslipidemia, sleep apnea syndrome, or obesity-induced cardiomyopathy) or had a BMI between 40 and 45 kg/m^2^ regardless of comorbidities. Participants with DM2 were limited to 10% of total enrollment so that the weight loss-limiting impact of diabetes would not have an undue impact on study results.

### 2.2. Study Design

The ReCharge trial is a 5-year, double-blind, Sham-controlled trial comparing vBloc to an implanted Sham device. Participants were randomized at implant in a 2 : 1 ratio to vBloc and Sham control arms in permuted block sizes of 3 or 6 stratified by clinical site and type 2 diabetes mellitus status.

Participants, sponsor personnel, and follow-up staff were blinded. The surgeon and the surgery support team could not be blinded, so their interaction with participants after randomization was limited until the 12-month blinded period of the trial had elapsed for all participants.

Subjects were treated in accordance with the Helsinki Declaration of 1975.

### 2.3. Intervention

Implant of the vBloc device and electrodes requires standard laparoscopic surgery performed under general anesthesia [5]. Electrodes are placed around the anterior and posterior vagal nerve trunks near the gastroesophageal junction, secured with sutures and connected to a rechargeable neuroregulator placed in a subcutaneous pocket on the thoracic side wall. The neuroregulator is recharged transcutaneously with a transmit coil placed over the neuroregulator connected to a mobile charger.

Participants randomized to the Sham arm were implanted with a similar neuroregulator that dissipated charge at a rate similar to the active device into a resistor within the Sham neuroregulator. Electrodes were not implanted and the vagal nerve trunks were not manipulated. To support the blind, Sham patients had the same number of skin incisions to simulate a laparoscopic procedure, but without entering the abdominal cavity. Active and Sham neuroregulators were sized identically at 8.6 cm in diameter, 7.1 cm in width, and 1.6 cm thick.

The neuroregulators in both groups were programmed to deliver therapy for at least 12 hours per day (though no therapy was delivered in the Sham group). Therapy energy levels were increased over the first month to the desired amplitude of 6 mA, although the amplitude could be adjusted by the follow-up team if the participant felt uncomfortable therapy-related sensations at any time during the trial. All monthly visits were collected within a ±2 week window. All participants were asked to check their battery level daily and to recharge their battery approximately twice weekly.

Follow-up visits occurred weekly in the first month, biweekly through month 3, and monthly thereafter through the second year. All participants participated in a weight management program that coincided with clinic visits. The weight management program typically consisted of a 15-minute educational interaction discussing healthy food choices, physical fitness, and social support. No specific diet (e.g., portion-controlled meals) or exercise program was prescribed.

### 2.4. Study Objectives

The 12-month primary efficacy and safety endpoints of the study have been previously reported [1]. Efficacy and safety continue to be assessed for 5 years. This report focuses on efficacy and safety at 18 months. Related serious adverse events, as defined in the previous publication [1], through 18 months were classified according to the Clavien-Dindo classification of surgical complications [6].

### 2.5. Statistical Analysis

Analyses of weight loss data were conducted under the intention-to-treat (ITT) principle using mixed-effects regression models [7]. Data were analyzed using a linear mixed model with unstructured covariance matrix, treating time (study visits) as a categorical variable with time-specific contrasts. Under this model, data are treated assuming missingness at random.

Since participants were unblinded on a rolling basis after 12 months, two sensitivity analyses were performed to ensure that weight-related trends were not attributable to effects of unblinding. Firstly, a mixed-effects model was fit with a time-varying covariate for unblinding and its interaction with the treatment group to test whether the treatment effect was affected by unblinding of participants. Secondly, the mixed-model was fit to the subset of data for which participants were still blinded.

In this report, results are not reported using the last observation carried forward (LOCF) method, the primary imputation method for the 12-month results [1], due to the poor statistical properties of LOCF imputation [7]. All statistical analyses were performed using SAS version 9.3.

## 3. Results

### 3.1. Baseline Characteristics and Participant Disposition

**Figure 1:**
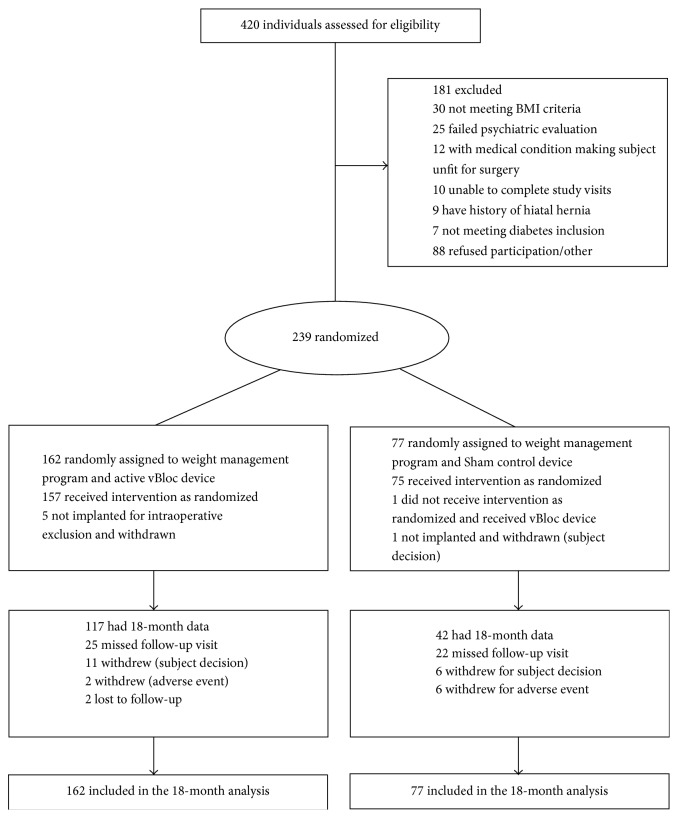
CONSORT diagram through 18 months.

The baseline characteristics of the ITT population are summarized in Table 1. A CONSORT diagram through 18 months is shown in Figure 1.

By the 18-month visit, 20 participants (12.3%) in the vBloc group and 13 participants (16.9%) in the Sham group had withdrawn from the study. Five withdrawals in the vBloc group and 1 in the Sham group occurred at implant and have been previously discussed [1]. After implant in the vBloc group, 2 participants were lost to follow-up, 3 participants withdrew for an adverse event (pain at the neuroregulator site, heartburn, and pain with therapy, resp.), and 10 withdrew for subject decision. In the Sham group, 6 withdrawals were for adverse events (2 for pain at the neuroregulator site, rotator cuff pain, irritable bowel syndrome, anxiety, and breast cancer, resp.) and 6 for subject decision.

The 18-month visit completion rates were 72% in the vBloc group and 55% in the Sham group; however, approximately 80% of participants in the ITT population in both groups had weight measurements within 2 months of the 18-month visit, which were incorporated in the statistical analysis of 18-month results. Eleven participants in the vBloc group and 19 in the Sham group who did not attend the 18-month visit had attended either their 16- or 17-month visit.

In addition to the revision procedures reported in the first 12 months [1], there were an additional 3 revisions in the vBloc group between 12 and 18 months. Two were for adverse events and one for a device malfunction. The revision procedures were uncomplicated and the patients were released within a day of the procedure.

### 3.2. Blinding

All participants and blinded study personnel remained blinded to randomization assignments until all participants had completed their 12-month visit and the 12-month study database was locked and verified. Since the study was enrolled over approximately 7 months, the majority of participants were not unblinded until their 16-month visit. At 15 months, 85% of subjects in the vBloc group and 90% of subjects in the Sham group remained blinded. At the 18-month visit, 27% of vBloc participants and 25% of Sham participants were still blinded.

### 3.3. Weight Loss

**Figure 2:**
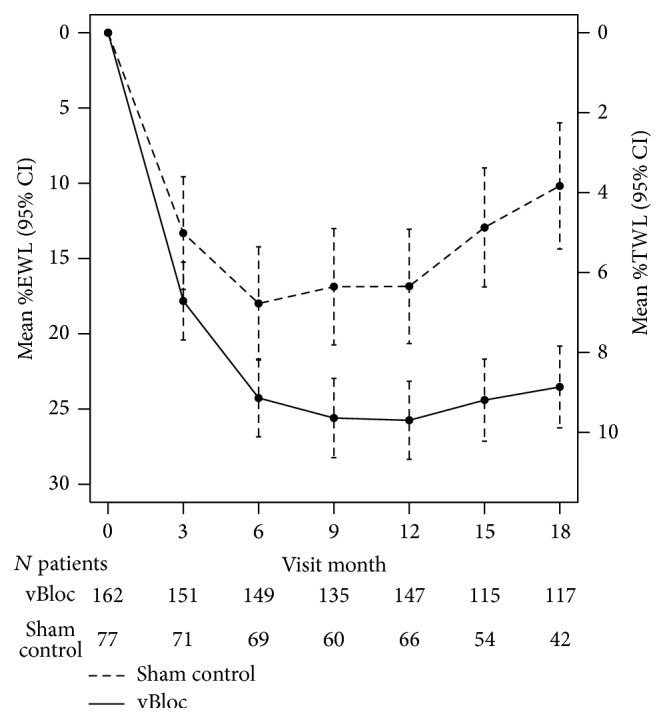


Weight loss as both %EWL and %TWL over time is shown in Table 2 and Figure 2. For the ITT population at 12 months, the estimated mean %EWL was 26% in the vBloc group and 17% in the Sham group (*P* < 0.001). At 15 months, where 86% of subjects remained under the study blind, the estimated mean %EWL was 24% for the vBloc group versus 13% for the Sham group (*P* < 0.001). At 18 months, the estimated mean %EWL was 24% for vBloc and 10% EWL for Sham (*P* < 0.001). The corresponding treatment difference between groups increased from 9 percentage points at 12 months (95% CI, 4–14) to 13 percentage points (95% CI, 8–18) at 18 months.

Sensitivity analyses showed that unblinding did not significantly influence the larger treatment effect with vBloc over time as the Sham group regained weight (*P* = 0.34 for the unblinding by treatment interaction). Similarly, when the analysis was restricted to patients who remained blinded throughout 18 months, the estimated mean %EWLs were similar to that of the overall sample. At 15 months, the estimated mean %EWL was 26% in vBloc and 13% in Sham (*P* < 0.001); at 18 months, the estimated mean %EWL was 21% in vBloc and 8% in Sham (*P* < 0.001).

Among the patients completing the 18-month visit (without imputation), the mean %EWL was 25% in the vBloc group and 12% in the Sham group (treatment difference, 13 percentage points; 95% CI, 6–21). At 18 months, 54% of vBloc patients achieved at least 20% EWL compared to 26% in the Sham group (*P* = 0.002); 41% vBloc patients achieved at least 25% EWL compared to 17% in the Sham group (*P* = 0.004).

### 3.4. Safety

The safety profile of vBloc remained favorable at 18 months. The adverse event (AE) profiles of both treatment groups were similar to that reported at 12 months [1]. All related AEs are shown in Table 3. The most commonly reported related AEs were heartburn and dyspepsia, abdominal pain, another pain, eructation/belching, and dysphagia. Ninety-eight percent of all AEs in the trial were reported as mild or moderate in severity (versus only 2% reported as severe) and 79% of events had resolved at 18 months. Most of the related events were transient side effects of therapy and resolved either spontaneously with no intervention or with an alteration of the therapy algorithm.

One additional surgical complication occurred between 12 and 18 months in the vBloc group. One patient had a gastric perforation at the gastroesophageal junction during explant of the device following the participant's decision to discontinue in the study. Following repair of the perforation, the patient improved postoperatively and fully recovered.

Table 4 shows the serious adverse events (SAEs) that occurred through 18 months classified according to the Clavien-Dindo Scale. This analysis demonstrates that 56% of the SAEs were grade I in severity, 6% were grade II (and this patient was not implanted but needed a transfusion due to bleeding from biopsy of a cirrhotic liver), 31% were grade III, and 6% was grade IV. Importantly, all patients had a full recovery without sequelae.

## 4. Discussion

Results of the ReCharge trial at the 18-month time point provide important context for weighing the benefits and risks of vBloc therapy. First, the trial continues to demonstrate sustained weight loss with vBloc therapy. Second, vBloc appears to have a favorable safety profile with a low risk of serious complications (0.6% of patients had a grade IV complication), and nonserious complications were typically mild or moderate sensations of the therapy that were resolved with little to no intervention. Interestingly, weight loss in the Sham group was considerably diminished within 6 months of the 12-month endpoint, despite continued blinding of the study past the 12-month visit and ongoing weight management counseling. These 18-month data were the topic of a meeting of the FDA Gastroenterology and Urology Devices Panel in June 2014 to consider US regulatory approval of the Maestro Rechargeable System to deliver vBloc therapy. The independent panel voted that the benefits of vBloc therapy outweighed the risks, and FDA subsequently granted approval to the Maestro Rechargeable System in January 2015.

Given that a large proportion of persons with moderate to severe obesity do not present for traditional bariatric surgical procedures secondary to the concerns for serious complications and permanent alteration of their gastrointestinal anatomy [4, 8], access to additional less-invasive options will be attractive to these individuals. While longer-term efficacy data are needed, the continued durability of weight loss with vBloc through 18 months provides additional support that vagal block may be considered an effective alternative to conventional weight loss surgery.

Weight loss in the Sham group was thought to result from a combination of Sham surgery including a Sham device, self-monitoring due to daily interaction with the Sham device to recharge the battery, and the weight management program [1]. Weight loss in the Sham group was more than expected, since participants were not prescribed a diet (e.g., portion-controlled meals) or physical activity, and as such, the primary 12-month objectives of the trial were not met. Sham surgeries in other contexts have also produced large effects [9–11], but these effects would be expected to be transient since no active treatment is being delivered. Surprisingly, weight loss in the Sham group of the ReCharge trial was relatively stable between 6 months and 12 months but deteriorated considerably thereafter. We suspect that this prolonged Sham effect may have resulted from enhanced self-monitoring due to the daily interaction with the Sham device.

Statistical modeling of the 18 month results suggest that unblinding of participants did not have a significant impact on the weight trajectories of either group and that weight regain in the Sham group occurred regardless of whether or not participants remained blinded. This 50% relative increase in the treatment effect of vBloc therapy through 18 months compared to 12 months indicates more substantial efficacy than that previously reported with vBloc compared to a rigorous Sham control.

Several limitations of the present report should be noted. First, frequency of missing data was appreciable at 18 months. However, nearly 80% of randomized participants in both groups had a visit within 2 months of the 18-month time point and all analyses were conducted on the ITT sample using all available longitudinal data, so inference can be made to the entire cohort at 18 months. Second, statistical analysis of the ReCharge study was not prespecified after 12 months. This limitation is offset by the continued analysis of the ITT cohort rather than a convenience sample as well as sensitivity analyses that concurred with the overall analysis. Finally, all participants were not blinded through 18 months and were unblinded on a rolling basis, making interpretation more difficult. However, nearly 90% of participants were still blinded through the 15-month visit, at which point much of the weight regain in the Sham group had already taken place. Additional analyses also suggest that unblinding did not impact weight regain in the Sham group.

## 5. Conclusions

Follow-up through 18 months of the ReCharge study showed sustained weight loss with intermittent vagal nerve block but not with a Sham surgery and device intervention. vBloc therapy continued to be safe and well tolerated. Additional long-term data and continued follow-up of the ReCharge study are needed to further characterize the safety and effectiveness profile of vBloc therapy.

## Figures and Tables

**Table 1:** Baseline characteristics by treatment group.

	vBloc	Sham control
	(*n* = 162)	(*n* = 77)
Demographics		
Women, number (%)	141 (87)	62 (81)
Age, mean (SD), y	47 (10)	47 (9)
Ethnicity, number (%)		
Caucasian	149 (92)	73 (95)
African American	8 (5)	3 (4)
Native American	2 (1)	1 (1)
Asian	1 (1)	1 (1)
Hawaiian/Pacific Islander	1 (1)	0 (0)
General medical		
Body size measures at implant, mean (SD)		
Height, cm	166 (8)	168 (9)
Implant weight, kg	113 (13)	116 (14)
BMI, kg/m^2^	41 (3)	41 (3)
Excess weight^a^, kg	44 (9)	45 (10)
Waist circumference, cm	121 (12)	123 (11)
Type 2 diabetes mellitus, number (%)	7 (4)	5 (7)
Hypertension, number (%)	63 (39)	32 (42)
Dyslipidemia, number (%)	91 (56)	46 (60)
Obstructive sleep apnea, number (%)	33 (20)	23 (30)

^a^Excess weight was calculated as the difference between the weight at the time of implantation and the ideal body weight corresponding to a BMI of 25 kg/m^2^.

**Table 2:** Mean %EWL and %TWL at 12, 15, and 18 months in ITT population.

Measure Visit month	Mean (95% CI)		Difference (95% CI)
	vBloc	Sham control	
	(*n* = 162)	(*n* = 77)	
%EWL			
12 months	25.8 (23.2, 28.4)	16.9 (13.1, 20.7)	8.9 (4.3, 13.5)
15 months	24.4 (21.7, 27.2)	12.9 (9.0, 16.9)	11.5 (6.7, 16.3)
18 months	23.5 (20.8, 26.3)	10.2 (6.0, 14.4)	13.4 (8.4, 18.4)
%TWL			
12 months	9.7 (8.7, 10.7)	6.4 (4.9, 7.8)	3.3 (1.6, 5.0)
15 months	9.1 (8.1, 10.1)	4.9 (3.4, 6.4)	4.2 (2.4, 6.0)
18 months	8.8 (7.8, 9.8)	3.8 (2.2, 5.4)	5.0 (3.1, 6.9)

**Table 3:** Cumulative adverse events related to device, procedure, or therapy through 18 months.

Adverse event	vBloc			Sham control		
	(*n* = 162)			(*n* = 77)		
	Number (%) of patients	Number of events	% events mild/moderate severity	Number (%) of patients	Number of events	% events mild/moderate severity
Pain, neuroregulator site	61 (38)	76	96%	32 (42)	36	100%
Heartburn/dyspepsia	41 (25)	45	100%	3 (4)	3	100%
Pain, other	40 (25)	48	96%	0 (0)	0	—
Pain, abdominal	22 (14)	30	100%	2 (3)	2	100%
Eructation/belching	14 (9)	14	100%	0 (0)	0	—
Dysphagia	13 (8)	13	100%	0 (0)	0	—
Chest pain	13 (8)	13	92%	2 (3)	2	100%
Nausea	12 (7)	17	94%	1 (1)	1	100%
Incision pain	12 (7)	14	100%	7 (9)	7	100%
Cramps, abdominal	8 (5)	8	100%	0 (0)	0	—
Wound redness or irritation	8 (5)	8	100%	5 (7)	5	100%
Bloating, abdominal	7 (4)	8	100%	1 (1)	2	100%
Constipation	7 (4)	7	100%	7 (9)	7	100%
Emesis/vomiting	6 (4)	8	88%	2 (3)	2	100%
Headache	6 (4)	6	100%	2 (3)	2	100%
Appetite increased	5 (3)	6	100%	2 (3)	3	100%

Only adverse events attributed by the investigator to the device, procedure, or therapy that occurred in at least 3% of vBloc group participants are displayed.

**Table 4:** Serious adverse events graded as surgical complications according to the Clavien-Dindo Scale through 18 months in the vBloc group.

Serious adverse event	vBloc		Grade	Rationale for grade
	Number (%) of patients	Number of events		
SAEs related to device, implant/revision, or therapy				
Neuroregulator malfunction	2 (1.2)	2	IIIb (2 events)	No abdominal surgery required, replacement with skin incision in same pocket
Atelectasis	1 (0.6)	1	I	Analgesics and antiemetics required with observation
Gallbladder disease	1 (0.6)	1	IIIb	Surgery for gallbladder removal required
Emesis/vomiting	1 (0.6)	1	IIIb	Surgery to reduce and repair hiatal hernia
Pain, neuroregulator site	1 (0.6)	1	IIIb	Skin incision, removal of fibrotic tissue, and pocket expansion required
Gastric perforation	1 (0.6)	1	IVa	Life-threatening complication with ICU management

SAEs related to intra-abdominal surgery				
Nausea	6 (3.7)	6	I (6 events)	Antiemetics and observation required
Cirrhosis^*∗*^	1 (0.6)	1	II	Transfusion required due to bleeding from liver biopsy
Generalized ileus	1 (0.6)	1	I	Analgesics required
Intraoperative oozing	1 (0.6)	1	I	Observation only

Total grades	Grade I: 9 events (5.6% of patients)			
	Grade II: 1 event (0.6% of patients)			
	Grade III: 5 events (3.1% of patients)			
	Grade IV: 1 event (0.6% of patients)			

^*∗*^Cirrhosis was found during the implant procedure and the patient was not implanted with a Maestro System.
